# Paving the way for the use of the SDQ in economic evaluations of school-based population health interventions: an empirical analysis of the external validity of SDQ mapping algorithms to the CHU9D in an educational setting

**DOI:** 10.1007/s11136-015-1218-x

**Published:** 2016-01-08

**Authors:** Nicole R. S. Boyer, Sarah Miller, Paul Connolly, Emma McIntosh

**Affiliations:** Health Economics and Health Technology Assessment, Institute of Health and Wellbeing, University of Glasgow, 1 Lilybank Gardens, Glasgow, G12 8RZ UK; Centre for Effective Education, School of Education, Queen’s University Belfast, Belfast, BT7 1LN UK

**Keywords:** SDQ, CHU9D, Child health utility, Health outcomes, School-based intervention, Population health

## Abstract

**Purpose:**

The Strengths and Difficulties Questionnaire (SDQ) is a behavioural screening tool for children. The SDQ is increasingly used as the primary outcome measure in population health interventions involving children, but it is not preference based; therefore, its role in allocative economic evaluation is limited. The Child Health Utility 9D (CHU9D) is a generic preference-based health-related quality of-life measure. This study investigates the applicability of the SDQ outcome measure for use in economic evaluations and examines its relationship with the CHU9D by testing previously published mapping algorithms. The aim of the paper is to explore the feasibility of using the SDQ within economic evaluations of school-based population health interventions.

**Methods:**

Data were available from children participating in a cluster randomised controlled trial of the school-based roots of empathy programme in Northern Ireland. Utility was calculated using the original and alternative CHU9D tariffs along with two SDQ mapping algorithms. *t* tests were performed for pairwise differences in utility values from the preference-based tariffs and mapping algorithms.

**Results:**

Mean (standard deviation) SDQ total difficulties and prosocial scores were 12 (3.2) and 8.3 (2.1). Utility values obtained from the original tariff, alternative tariff, and mapping algorithms using five and three SDQ subscales were 0.84 (0.11), 0.80 (0.13), 0.84 (0.05), and 0.83 (0.04), respectively. Each method for calculating utility produced statistically significantly different values except the original tariff and five SDQ subscale algorithm.

**Conclusion:**

Initial evidence suggests the SDQ and CHU9D are related in some of their measurement properties. The mapping algorithm using five SDQ subscales was found to be optimal in predicting mean child health utility. Future research valuing changes in the SDQ scores would contribute to this research.

## Background

The importance of children’s social and emotional well-being (SEW) is gaining increased attention in educational and policy circles with growing evidence linking early SEW to later academic performance and various health outcomes including mental health [1–3]. Research suggests social–emotional competency at a young age is associated with increased well-being and school performance, while problems with these competencies can lead to personal, social, and academic difficulties [4, 5]. Children with emotional and behavioural problems are more likely to develop mental health disorders (which predict social mobility and unemployment) [6], be involved in crime or violence [7], practice unsafe sex, and misuse drugs and alcohol [8]. Increased interest exists in the role of school-based programmes to improve SEW as a means to promote children’s successes in school and life. A recent meta-analysis of school-based social and emotional learning programmes found participants to have significantly improved social and emotional skills, attitudes, behaviour, and academic performance [9]. Effects diminished in follow-up, but remained statistically significant for 6 months after intervention [9]. Few studies report follow-up longer than 6 months [9], and long-term effectiveness and cost-effectiveness of these programmes are uncertain. The long-term broader impacts of school-based SEW programmes on educational outcomes, health behaviours, adult unemployment, crime, and health-related outcomes are important to identify as these potential impacts inform any comprehensive economic evaluation of SEW programmes.

Roots of Empathy (RoE) was developed in Canada with aims of increasing empathy, prosocial behaviour and decreasing aggressive behaviour in children [10]. At the heart of the programme is the development of empathy among children. RoE consists of 27 lessons based on a monthly visit from an infant and parent whom the class ‘adopts’ at the beginning of the school year.

A characteristic of RoE is that it is a mentalisation-based programme. Mentalisation is the ability to focus on mental states in oneself and others to understand behaviour [11]. The labelling of feelings and exploration of the relationship between feelings and behaviour is achieved through observation of the mother–infant interaction in the classroom. Clearly, the baby cannot communicate in words and can only express his/her feelings through behaviour. For this reason, the baby in RoE provides an ideal opportunity for children to learn mentalisation skills through interpreting and labelling the baby’s emotions. They learn affective and cognitive components of empathy, enabling them to empathise with others.

The Strengths and Difficulties Questionnaire (SDQ) is a behavioural screening tool which has been widely validated and used in a number of studies internationally [12]. The 25-item behavioural and emotional assessment tool is shorter than other instruments such as the Child Behaviour Checklist [13]. The SDQ is also less dated with a focus on identifying children’s strengths rather than focusing on their deficits as with the traditional yet well-established Rutter Questionnaire [14]. The SDQ consists of five symptom scales (emotional, conduct problems, hyperactivity, peer problems, and prosocial behaviour) with five items each. A further sixth scale, total difficulties, is the sum of symptom scale scores excluding prosocial behaviour. It is clear the SDQ is a favoured primary outcome measure of SEW in school-based interventions; however, due to its measurement properties, (i.e. lack of a value-based outcome) its applicability in economic evaluation is limited. The SDQ has been widely used in Child and Adolescent Mental Health Services (CAMHS) throughout the UK [15] providing routinely collected data that could be readily translated into health utilities (via appropriate means), thus providing an additional tool for the facilitation of economic evaluation; however, its use and applicability for economic evaluations within a school-based context is under-researched.

Measuring SEW in a school environment is highly challenging as it is recognised that a lack of valid methods exists for primary school children [3]. A recent review of eleven mental health outcome measures found none to have sufficient psychometric evidence to reliably measure severity and change over time in key groups [16]. Despite this, the use of the SDQ [17] has been viewed positively by staff in preschool establishments [18] and is recently being used in school-based settings to assess SEW [19–22]. There is an added need for research into measuring the effectiveness of SEW interventions outwith health care CAMHS settings and within the school context, in particular how SEW is valued within cost–utility analysis of school-based interventions, which tools are best placed to do this valuing, and how these short-term outcomes translate to meaningful long-term outcomes within economic evaluations.

Measuring and valuing health-related quality of life (HRQoL) is fundamental in determining the cost-effectiveness of health improvement programmes such as RoE [23]. Where such interventions have a claim on society’s scarce resources, their worthwhileness must be evidenced so as to ensure optimal allocation of resources. HRQoL instruments are generally categorised into two groups, preference and non-preference based [24]. During development, HRQoL items or attributes in the former are weighted by the populations’ preferences using methods such as the standard gamble (SG), time-trade-off (TTO), or ranked or scaling methods [25]. In the area of child health, however, the latter is more widely used due to a lack of validated preference-based measures of quality of life (QoL) for children [26]. Preference-based measures such as the EuroQol EQ-5D [27] and Health Utilities Index (HUI) [28] are generic, can be applied over a range of disease and population areas, and can be used in calculation of quality-adjusted life years (QALYs) to facilitate cost–utility analysis.

An advantage of using preference-based measures is that their descriptive systems have been valued, so changes in health states can be directly linked to utility values. Utilities are cardinal values that represent individuals’ preferences for health states. Instruments such as EQ-5D typically measure utility on a scale between 0 and 1 where 0 represents death and 1 represents full health. The National Institute for Health and Care Excellence (NICE), a decision-making body in the UK, recommends QALYs as the preferred measure of health outcome. Resource allocation decisions include cost-effectiveness criteria with a willingness-to-pay threshold of around £20,000–£30,000 per QALY. Non-preference-based measures such as the Paediatric Quality of Life Inventory (PedsQL) [29] and Child Health Questionnaire (CHQ) [30] exist for measuring HRQoL in children; however, because they are not preference-based, they cannot be used in cost–utility analysis. Another problem with these types of measures is that they usually have separate scores for different domains; so it can be difficult to value overall change if some domains show improvements, while others deteriorate.

Two key challenges exist when performing economic evaluations of paediatric population health interventions: (1) lack of suitable preference-based outcome measures for all age ranges and (2) importance of, and requirement for, longer-term extrapolation of multi-sector costs and benefits. The NICE preferred measure of HRQoL is EQ-5D [31], which was developed for adults. A youth version was derived from the adult version, the EQ-5D-Y; however, there is debate over the appropriateness of using adult preference-based measures to derive paediatric QALYs and more generally, whose values are relevant in economic evaluation [32]. Moreover, the existing social value sets for EQ-5D are not appropriate preference weights for paediatric populations [33]; thus, this missing value set is a limitation to use of the EQ-5D-Y in economic evaluation. The use of adult preference-based measures may not be appropriate for children and adolescents, and direct elicitation methods such as the SG or TTO pose challenges due to age, ethical, and cognitive limitations [34].

The Child Health Utility 9D (CHU9D) is a generic preference-based HRQoL instrument suitable for use with children ages 7–17 [35–37]. Qualitative and quantitative research was undertaken with children during its development to identify and assess dimensions of HRQoL and ensure the measure is child-centred [35, 38]. It has demonstrated itself as a practical and valid measure for use in economic evaluation of child and adolescent health care programmes [36, 37]. The CHU9D consists of nine dimensions: worried, sad, pain, tired, annoyed, school work, sleep, daily routine, and ability to join in activities, with five levels each. Each level of the nine dimensions is scored from 1 to 5, 1 representing perfect health and 5 the worst health state.

Two value sets containing preference weights for each health state are currently available as valuation of the CHU9D was directly elicited from both adult and adolescent populations. The original tariff was derived from 300 members of the UK adult population using a SG technique [35, 39]. Subsequently, an alternative tariff was developed; preference weights were derived from Best–Worst Scaling (BSW) discrete choice experiment interviews of 590 Australian adolescents aged 11–17 [40]. These value sets allow calculation of QALYs in economic evaluation of paediatric programmes. Additionally, two algorithms are available to predict mean group utility from three and five subscales of the SDQ [41].

NICE has developed separate guidance for technology appraisals of public health interventions recognising the differences in the nature and scope of population-based interventions [42]. The public health reference case encourages a broader perspective in economic evaluation with methods such as cost–consequence analysis and cost–benefit analysis (CBA). In CBA, health and non-health outcomes are valued in monetary terms which address the allocative efficiency question of whether a new programme such as RoE is a worthwhile programme to invest in, given the alternative health and well-being outcomes which could be achieved from use of classroom resources. Methodological challenges arise when considering how to capture these broader, multi-sector costs and benefits, and how these might be extrapolated over the lifetime of a child. Use of non-traditional economic outcomes such as the SDQ may provide a useful starting point for health economists as it is now established in long-term cohort studies [43, 44] as well as being recently mandated for use in Australia’s specialised CAMH services as a consumer-oriented outcome assessment tool. Furber et al. [41] outlines that national and international data coordination efforts (e.g. [45, 46]) have led to the creation of large SDQ datasets, which represent thousands of episodes of care in CAMH services across Australia and the UK. Transforming SDQ scores to utility values would facilitate cost–utility analyses of not only routine CAMHS data but would open up school-based SDQ data to the possibility of economic evaluation.

This study aims to contribute to the outcomes’ evidence base for economic evaluation of school-based population health interventions by testing and validating previously published mapping algorithms [41] to translate SDQ scores to utility values. Given this aim, our research question asks, ‘can SDQ scores elicited within an educational context be mapped using published algorithms to preference-based CHU9D utilities with a view to incorporating such utilities within an economic evaluation framework?’ An economic evaluation has been designed alongside the National Institute for Health Research funded RoE cluster randomised controlled trial evaluation in Northern Ireland (International Standard Randomised Controlled Trial Number Register: ISRCTN07540423). Primary outcome measures collected for the economic evaluation are the SDQ and CHU9D. Utility mapping methods have been conducted to transform SDQ scores into CHU9D values [41]; beyond that, we are unaware of any completed economic evaluations using these two measures together or indeed externally validating the algorithms. Use of preliminary non-randomised data from the RoE trial provided a unique opportunity to explore the relationship between these two measures as well as externally validate the SDQ mapping algorithm developed by Furber et al. [41].

## Methods

### Study population

The RoE programme was aimed at primary five pupils (aged 8–9 years). Seventy-four primary schools were recruited from four of the five trusts in Northern Ireland. Data were collected from 67 schools (*n* = 1179) at baseline (October 2011), 65 schools (*n* = 1181) after intervention completion (June 2012), and 64 schools (*n* = 1277) at 12-month follow-up (June 2013). Schools were randomly allocated to either the intervention group which received RoE during the 2011–2012 academic year or the control group which continued with usual curriculum.

### Data collection

Teachers completed the SDQ for each participating child at each time point. The teacher complete version is a proxy for child behaviour outcome, as a self-complete version is available for older children aged 11–17. Experienced fieldworkers visited schools and administered CHU9D questionnaires to the whole class. Children were asked not to confer, and this was ensured by the fieldworker and class teacher. Each question was read aloud to the class, and any words or phrases that were difficult were explained. Consent forms were sent home with children prior to baseline data collection. Deprivation was measured by the Northern Ireland Multiple Deprivation Measure 2010 (NIMDM) which is a relative measure of deprivation [47].

### Outcome measures

#### Strengths and Difficulties Questionnaire

The primary outcome measure for the trial was the SDQ. There are three forms of the questionnaire available: teacher complete (ages 4–17), caregiver complete (i.e. legal parent or guardian) (ages 2–4 and 4–17), or self-complete by the pupils (ages 11–17) [12]. The teacher complete proxy version was used.

The SDQ was scored using the predictive algorithm converted into Stata syntax available on the SDQinfo website [12] in Stata 11.2 (StataCorp LP, College Station, Texas, USA). This involved assigning a score from 0 to 2 (0 = no difficulties, 2 = many difficulties) for each item of the questionnaire and summing the total for each scale. Totals from all scales (excluding prosocial behaviour) were then summed to generate the total difficulties score.

SDQ scores can be classified into four bands that reflect the general population; these bandings were based on a large UK community sample provided elsewhere [48]. The bandings categorise SDQ scores into four groups: ‘close to average’ (80 % of the population), ‘slightly raised’ (10 %), ‘high’ (5 %), and ‘very high’ (5 %). The teacher complete four-band categorisation for SDQ scores is given below in Table 1.

**Table 1:** SDQ domain score four-band categorisation

Teacher complete	Close to average	Slightly raised/lowered	High/low	Very high/very low
Total difficulties score	0–11	12–15	16–18	19–40
Emotional problems score	0–3	4	5	6–10
Conduct problems score	0–2	3	4	5–10
Hyperactivity score	0–5	6–7	8	9–10
Peer problems score	0–2	3–4	5	6–10
Prosocial score	6–10	5	4	0–3

From http://www.sdqinfo.org/py/sdqinfo/b3.py?language=Englishqz(UK) scoring instructions for SDQs for 4- to 17-year-olds

### Child Health Utility 9D

There are two value sets available for the CHU9D: (1) the original tariff where preference weights were obtained from a general UK adult population using SG technique, and (2) the alternative tariff where preference weights were obtained from an Australian adolescent population using BWS. Each value set was applied to CHU9D scores to calculate utility values, for comparative purposes. For the original tariff (SG), coefficients from the ordinary least squares (OLS) parsimonious model (model 5) [35] were used as decrements to calculate utility. For the alternative tariff (BWS), rescaled conditional logit estimates were used [40].

Two OLS regression-based algorithms [41] were applied to transform SDQ scores into utility values. These regressions were previously developed by running CHU9D utility values as the dependent variable and SDQ subscales as predictors. In this study, both measures were assessed by parent proxy, which differs from the currents study where SDQ is assessed by teacher proxy and CHU9D by children themselves. Both algorithms using three and five SDQ subscales are replicated in (1) and (2) below from Furber et al. [41].

1. Algorithm using five SDQ subscales [41]

$$\begin{aligned} {\text{Utility}} & = 0.880 + \left( { - 0.019 \times {\text{emotion}}} \right) + ( - 0.009 \\ & \quad \times {\text{conduct)}} + \left( { - 0.001 \times {\text{hyper}}} \right) + ( - 0.008 \\ & \quad \times {\text{peer)}} + \left( {0.005 \times {\text{prosocial}}} \right) \\ \end{aligned}$$

2. Algorithm using three SDQ subscales [41]

$$\begin{aligned} {\text{Utility}} & = 0.918 + \left( { - 0.018 \times {\text{emotion}}} \right) \\ & \quad + \left( { - 0.012 \times {\text{conduct}}} \right) + \left( { - 0.009 \times {\text{peer}}} \right) \\ \end{aligned}$$

### Analysis

Missing data were modelled through multiple imputation (MI) via chained equations as recommended by good research practice guidelines [49–52]. As both CHU9D and SDQ responses are ordered categorical variables, an ordinal logistic regression model was selected. Descriptive statistics [mean, standard deviation (SD)] were generated for gender, grade level, deprivation rank, and each scale of the SDQ and CHU9D. Tables of frequency are graphed for CHU9D and SDQ level responses for a visual representation of the spread and nature of the data. When assessing the agreement between prosocial behaviour, total difficulties, and utility measures, variables were plotted in pairs to check for approximate linearity, outliers and subgroups. Normality was assessed using a Skewness/Kurtosis test. It is hypothesised that utilities will be non-normal, but due to the large sample size the normality assumption can be overlooked. Pearson’s correlation coefficient was used to assess the strength of relationship between utility, total difficulties, and prosocial behaviour. *t* tests were performed to test for pairwise differences in utility values created from original tariff [35], alternative tariff [40], and both mapping algorithms [41].

## Results

Questionnaires were returned by teachers in 67 schools at baseline, 65 schools after intervention, and 64 schools at 12-month follow-up. The three schools that dropped out came from a range of different types and deprivation levels, so it is unlikely that they would bias results. After data cleaning and MI, a total of 1254 child participants were included in the analysis making up 3762 observations. At baseline, a majority of the pupils (88.9 %) were recruited in Primary 5 (approximately 9 years old); however, some Primary 4 and Primary 6 pupils were also included. Table 2 presents the characteristics of these participants. The sample was made up of 51.5 % boys, and median deprivation rank was 430 which is comparable to median population rank of 445. As the sample deprivation rank is less than the median rank, it can be said the sample median is more deprived than the population median rank, but the extent to which the sample is more deprived cannot be inferred from the rankings.

**Table 2:** Characteristics of participants

Characteristics	Participants^a^ (*n* = 1254)	British community sample^b^
Gender		
Boys, *n* (%)	646 (51.5)	
Girls, *n* (%)	608 (48.5)	
Grade level		
P4 (≈8 years old), *n* (%)	81 (6.5)	
P5 (≈9 years old), *n* (%)	1115 (88.9)	
P6 (≈10 years old), *n* (%)	58 (4.6)	
NIMDM deprivation rank^c^, median (SD)	430 (245.9)	
SDQ total difficulties, mean (SD)	12 (3.2)	6.6 (6.0)
SDQ prosocial subscale, mean (SD)	8.3 (2.1)	7.2 (2.4)
SDQ emotion subscale, mean (SD)	1.5 (2.0)	1.4 (1.9)
SDQ conduct subscale, mean (SD)	2.3 (1.0)	0.9 (1.6)
SDQ hyperactivity subscale, mean (SD)	4.1 (1.3)	2.9 (2.8)
SDQ peer problems subscale, mean (SD)	4.1 (0.9)	
CHU9D original tariff, mean (SD)	0.84 (0.11)	
CHU9D alternative tariff, mean(SD)	0.80 (0.13)	
CHU9D algorithm using five SDQ subscales, mean(SD)	0.84 (0.05)	
CHU9D algorithm using three SDQ subscales, mean(SD)	0.83 (0.04)	

^a^Participants had responses at 3 time points for a total of 3762 observations

^b^From British sample 8208 teachers of children aged 5–15 http://www.sdqinfo.org/norms/UKNorm1.pdf

^c^Lower rank = higher deprivation

**Fig. 1:**
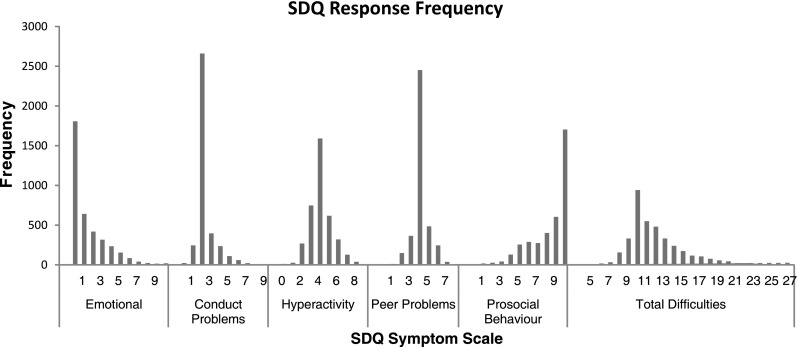
Frequency of strengths and difficulties questionnaire responses

The mean (SD) for SDQ total difficulties and prosocial behaviour scores was 12 (3.2) and 8.3 (2.1), respectively, which are classified as ‘slightly raised’ and ‘close to average’. The mean (SD) for SDQ subscales emotion, conduct, hyperactivity, and peer problems was 1.5 (2.0), 2.3 (1.0), 4.1 (1.3), and 4.1 (0.9). As a point of reference, the mean (SD) of SDQ subscales of a large community sample is provided in Table 2. Emotion and hyperactivity subscales were classified as ‘close to average’, and conduct and peer problems were ‘slightly raised’. The frequency of responses for each symptom scale is reported in Fig. 1.

**Fig. 2:**
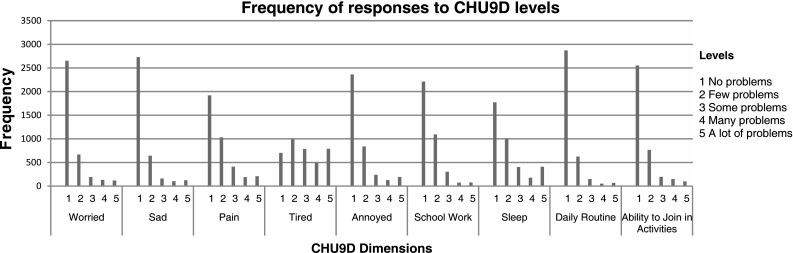
Frequency of child health utility 9D responses

The mean (SD) utility scores were 0.84 (0.11) and 0.80 (0.13) based on the original and alternative tariffs. These scores are commensurate with reported population health utility values [39, 53]. With both scoring algorithms, approximately 5.72 % of participants were classified in full health (i.e. utility = 1). In all dimensions of the CHU9D except ‘tired’, no problems were most commonly reported. Figure 2 reports the frequency of responses to all levels.

The mean (SD) utility values for the mapping algorithms using five and three of the SDQ subscales were 0.84 (0.05) and 0.83 (0.04). Each method for calculating utility produced statistically significantly different results except the original tariff and mapping algorithm using five SDQ subscales in which no statistically significant difference was detected (*p* = 0.69) (95 % CI −0.003, 0.004). Table 3 reports these differences.

**Table 3:** Differences in utility values

Difference in pair	*n*	Mean	SD	*t*	*df*	*p*	95 % CI
Original versus alternative	3762	0.036	0.051	43.926	3761	0.000	0.035, 0.038
Original versus 5 SDQ subscales	3762	0.001	0.116	0.402	3761	0.688	−0.003, 0.004
Original versus 3 SDQ subscales	3762	0.010	0.115	5.360	3761	0.000	0.006, 0.014
Alternative versus 5 SDQ subscales	3762	−0.036	0.136	−16.10	3761	0.000	−0.040, −0.031
Alternative versus 3 SDQ subscales	3762	−0.026	0.135	−12.022	3761	0.000	−0.031, −0.022
5 SDQ versus 3 SDQ subscales	3762	0.009	0.011	53.209	3761	0.000	0.009, 0.010

There were low, but statistically significant correlations between all combinations of CHU9D (original tariff), total difficulties, and prosocial behaviour. Pearson’s rank correlation coefficient showed significant correlations between: total difficulties and CHU9D (*r* = −0.08, *p* < 0.01), total difficulties and prosocial behaviour (*r* = −0.27, *p* < 0.01), and prosocial behaviour and CHU9D (*r* = 0.04, *p* = 0.02).

## Discussion

In this sample, half of teacher-rated SDQ subscales scores were ‘close to average’ and half were ‘slightly raised’. Total difficulties, conduct, and peer problems were classified as ‘slightly raised’ in comparison with a large UK sample [48]. Sample mean scores in each subscale were higher (indicating more difficulties) than UK average, except in prosocial behaviour where the sample mean was higher (indicating greater prosocial behaviour) [54, 55]. In terms of economic evaluation, this outcome on its own is less useful because the ‘value’ associated with unit changes in SDQ scores is unknown. For CHU9D, the majority of the sample was in the ‘no problems’ category, with the exception of ‘tired’ (see Fig. 2). With these differences between the two measures, there does not seem to be large overlap between descriptive systems. This is due to differences on a conceptual basis; the SDQ is a behavioural screening tool designed to assess emotional and behavioural functioning, while the CHU9D assesses the child’s broader functioning and HRQoL. However, when comparing single dimensions of the two measures in terms of frequency of responses (see Figs. 1, 2), there is some overlap. Worried and Sad dimensions of the CHU9D overlap the Emotional symptom scale of the SDQ well.

It is also important to note that despite all of the correlations between the SDQ and CHU9D being significant they were not very high; the statistical correlation may simply be a result of the large sample size. The SDQ alone cannot provide insight into resource allocation decision-making, and whether the programme is a worthwhile use of educational resources (or indeed an argument for investing health care resources). Yet, the SDQ is a common primary outcome measure in many paediatric population health interventions. For economic evaluation, the CHU9D is useful because it has value associated with incremental change.

The mean utility generated for the original tariff CHU9D was 0.84 which compares with the range of mean values reported in previous studies (0.803–0.86) [24, 56, 57]. The studies varied in context, setting, and age groups, but were included for comparison as so few studies have published CHU9D outcomes. The mean utility from alternative tariff CHU9D was lower than the original tariff which is consistent with recent Chinese and Australian studies that applied both tariffs to their samples [24, 58]. Ratcliffe and colleagues [58] have compared the adult (original) and adolescent (alternative) tariffs using the responses to a web-based survey of 500 Australian adolescents, aged 11–17. They found differences in adult and adolescent values for identical health states may have enough significance to impact on health care policy [58]. Differences between the instruments may be due to differences in descriptive systems, size and nature of the samples, and the valuation methods used to develop each scoring algorithm [58]. Nevertheless, the Chinese version CHU9D found utilities generated discriminated well in relation to self-reported health status, regardless of which value set was employed [24]. By applying the mapping algorithms to an external dataset, this research contributes to the existing evidence base around the suitability of the use of the five SDQ subscale mapping algorithm for eliciting utilities.

### Strengths and limitations

The advantage the CHU9D brings to the evaluation of paediatric interventions is that they can now be assessed using a preference-based measure, combined with costs and judgements made in relation to their relative cost-effectiveness. It is now possible to compare paediatric programmes from a range of areas that aim to improve different aspects of children’s health and well-being by including a measure such as CHU9D. Changes in effectiveness as measured using the SDQ and mapped to CHU9D can now be readily compared in terms of their costs required to achieve those changes in outcomes. For example, a cost per three-point change in the SDQ could not readily be compared to a cost per three-point increase on a national examination. Having a uniform measure of QoL that has been valued by the population allows comparison of programmes in terms of both costs and effects as they have been measured on the same generic scale.

To our knowledge, this is the first study to apply the preliminary mapping algorithms [41] to an external dataset. The caregiver version of the SDQ was used in development of these algorithms as opposed the teacher-rated version used in the current study. Additionally, parent-completed proxy report CHU9D was used [41], as opposed to child-completed CHU9D in the current study. This is a limitation as the validity of applying the mapping algorithms to different versions of SDQ and CHU9D is questioned (i.e. the validity of mapping from parent complete SDQ to child complete CHU9D). However, the CHU9D was intended to be completed by children, and our current sample was too young to fill in the child complete version of the SDQ (intended for ages 11–17).

Utilities derived from the four different approaches were all significantly different, and the only pair that was not was the original tariff and five SDQ subscale algorithm. This is an interesting finding because the population from which the algorithm was developed was sampled from CAMHS. These children would be expected to have lower QoL than a general school-aged population. Also, these algorithms were developed using the alternative tariff, and it is of note that in our results the five SDQ subscale algorithm better predicts the original tariff. Nonetheless, this study adds to the evidence and generalisability of the mapping algorithm using all five of the SDQ subscales [41].

Economic evaluation is now feasible in studies where SDQ data (but not utility data) have been collected and our results suggest the algorithm containing all five SDQ subscales to be superior. This is in line with recommendations [41]; however, future studies should replicate use of these algorithms to confirm these results.

The use of mapping to derive generic preference-based indices from disease specific measures raises a fundamental concern as mapping methods assume overlap in each measure’s descriptive systems [59]. Stronger mapping functions will have greater overlap between the descriptive systems. One method for assessing these functions is to evaluate the difference between predicted and observed values by calculating the root-mean-square error (RMSE) [59]. The RMSE gives an indication of the size of the prediction errors between predicted and observed values. With the mapping algorithms [41], RMSE indicated large differences between predicted and observed values at the individual level. However, the purpose of mapping methods is to predict differences across groups or between trial arms, not at the individual level. Due to the lacking overlap between the SDQ and CHU9D descriptive systems, the use of the mapping algorithm is a second best option to the use of preference-based HRQoL measures, but it may be necessary in population health programmes for pragmatic reasons.

This study has demonstrated initial evidence for the use of the SDQ in economic evaluation of school-based interventions. In broader settings outside of the adult health care sector (i.e. education, paediatric, and population health), industry-specific primary outcome measures such as the SDQ may be the only measure of effect collected. In these instances, this study indicates the five SDQ subscale algorithm as a useful instrument, affording health economists’ the opportunity to conduct preferred cost–utility analyses.

## Conclusion

The SDQ and CHU9D are able to measure outcomes in children aged 8–13 years within a school-based setting, and there is initial evidence that they are related in their measurement properties. When conducting economic evaluation of population-based interventions where traditional utility measurement methods are missing, preliminary findings suggest the mapping algorithm using five SDQ subscales optimal for predicting mean utility. This allows analysts the opportunity to conduct cost–utility analysis in paediatric or school-based programmes where previously this would have been challenging due to a lack of preference-based outcome measures. To our knowledge, the SDQ and CHU9D have not yet been used to predict longer-term outcomes within an economic evaluation context. This is an important avenue for further research as issues remain as to how these childhood measures extrapolate into adulthood.

## Abbreviations

SEW: Social and emotional well-being
RoE: Roots of Empathy
SDQ: Strengths and Difficulties Questionnaire
UK: United Kingdom
HRQoL: Health-related quality of life
SG: Standard gamble
TTO: Time trade-off
QoL: Quality of life
HUI: Health Utilities Index
QALY: Quality-adjusted life year
PedsQL: Paediatric Quality of Life Inventory
CHQ: Child Health Questionnaire
NICE: National Institute for Health and Care Excellence
CHU9D: Child Health Utility 9D
BWS: Best–worst Scaling
CBA: Cost–benefit analysis
GEE: Generalised estimating equations
NIMDM: Northern Ireland Multiple Deprivation Measure
MI: Multiple imputation
SD: Standard deviation
RMSE: Root-mean-square error
